# The emerging roles and therapeutic potential of B cells in sepsis

**DOI:** 10.3389/fphar.2022.1034667

**Published:** 2022-11-08

**Authors:** Chengyong Ma, Hanrui Liu, Shuo Yang, Hong Li, Xuelian Liao, Yan Kang

**Affiliations:** ^1^ Center of Translational Medicine, Key Laboratory of Birth Defects and Related Diseases of Women and Children, Ministry of Education, West China Second University Hospital, Sichuan University, Chengdu, China; ^2^ Department of Critical Care Medicine, West China Hospital, Sichuan University, Chengdu, China; ^3^ Development and Related Diseases of Women and Children Key Laboratory of Sichuan Province, West China Second University Hospital, Sichuan University, Chengdu, China

**Keywords:** B cell, sepsis, inflammation, innate immune, adaptive immune, therapeutic target

## Abstract

Sepsis is a life-threatening syndrome caused by anomalous host response to infection. The pathogenesis of sepsis is complex, and immune dysfunction is the central link in its occurrence and development. The sepsis immune response is not a local and transient process but a complex and continuous process involving all major cell types of innate and adaptive immunity. B cells are traditionally studied for their ability to produce antibodies in the context of mediating humoral immunity. However, over the past few years, B cells have been increasingly recognized as key modulators of adaptive and innate immunity, and they can participate in immune responses by presenting antigens, producing cytokines, and modulating other immune cells. Recently, increasing evidence links B-cell dysfunction to mechanisms of immune derangement in sepsis, which has drawn attention to the powerful properties of this unique immune cell type in sepsis. Here, we reviewed the dynamic alterations of B cells and their novel roles in animal models and patients with sepsis, and provided new perspectives for therapeutic strategies targeting B cells in sepsis.

## 1 Introduction

Sepsis occurs when infection exceeds local tissue containment and induces a series of maladaptive immune responses, leading to multiple organ dysfunction (Delano and Ward, 2016; Seymour et al., 2016; Singer et al., 2016). As a global disease with high morbidity and mortality, sepsis is a serious threat to human life, health, and safety (Rudd et al., 2020). Some patients with sepsis progress to septic shock, manifested by severe microcirculatory dysfunction and cellular metabolic disturbance, which is associated with higher mortality. With advances in intensive care medicine and intervention therapies, mortality rates in the early stages of sepsis have declined (Rudd et al., 2020). However, many uncontrolled inflammatory responses and intractable immunosuppression persist in the late stages of sepsis, leading to subsequent deaths of survivors from recurrent nosocomial and secondary infections (Rubio et al., 2019; Jarczak et al., 2021).

The pathogenesis of sepsis is a result of a complex network of events involving immune-inflammatory and anti-inflammatory processes triggered by pathogens including organisms such as bacteria, fungi, parasites, and viruses (Jarczak et al., 2021). After infection, the innate immune cells, such as neutrophils and macrophages, recognize pathogen-derived molecular patterns recognize and/or endogenous host-derived danger signals through an assortment of pattern recognition receptors (Huang et al., 2019). In most cases, the innate immune system could effectively eliminate the invading pathogen by releasing both pro- and anti-inflammatory mediators (van der Poll et al., 2017). However, some infections override local tissue containment and induce a series of dysregulated physiologic responses, which then become unbalanced and harmful to the host, ultimately leading to the development of sepsis (van der Poll et al., 2017). The most recent definition of sepsis is a life-threatening organ dysfunction that is caused by a dysregulated host response to infection (Singer et al., 2016). The new definition shifts the focus of sepsis pathophysiology to immune dysfunction, manifested by immunosuppression, chronic inflammation, and persistence of bacteria, which ultimately significantly alters patients’ innate and adaptive immune responses (van der Poll et al., 2021). Immune dysfunction in sepsis often leads to changes in the function and fate of immune cells, including immune cell exhaustion, maturation impairment, increased apoptosis, increased expression of inhibitory immune receptors, and increased regulatory T lymphocytes (Cao et al., 2019; van der Poll et al., 2021). To date, the changes and contributions of T cells and NK cells in the context of sepsis have been extensively studied. However, the alterations and dysfunction of B cells in sepsis are still poorly understood (Oltean, 2020).

B cells are traditionally known for their ability to secrete antibodies in the context of mediating adaptive immune responses. However, in addition to producing antibodies, B cells can participate in immune responses and exert vital immunoregulatory functions by presenting antigens, secreting an array of cytokines, and regulating the functions of other immune cells (Sage et al., 2019; Adamo et al., 2020). As a bridge between humoral and cellular immunity, B cells play an important role in the pathogenesis of a variety of human diseases, such as cardiovascular diseases, immune-mediated kidney diseases, and central nervous system diseases (Oleinika et al., 2019; Sabatino et al., 2019; Sage et al., 2019; Adamo et al., 2020). Recent evidence has linked B-cell dysfunction to sepsis, that is, B cell-deficient or anti-CD20-depleted mice exhibit exacerbated disease and reduced survival after bacterial sepsis, whereas mice deficient in marginal zone B cells have attenuated inflammation and prolonged survival in endotoxic shock (Kelly-Scumpia et al., 2011; Honda et al., 2016; Aziz et al., 2018). These data shed light on the complex role of B cells in sepsis and that they may aggravate or improve sepsis outcomes. This review focuses on summarizing the latest research on the changes in B cells and their emerging roles in sepsis. We also briefly summarize the cell biology of B cells and further discuss the application and therapeutic strategies targeting B cells in sepsis.

## 2 B Cells and their immunological function

**FIGURE 1:**
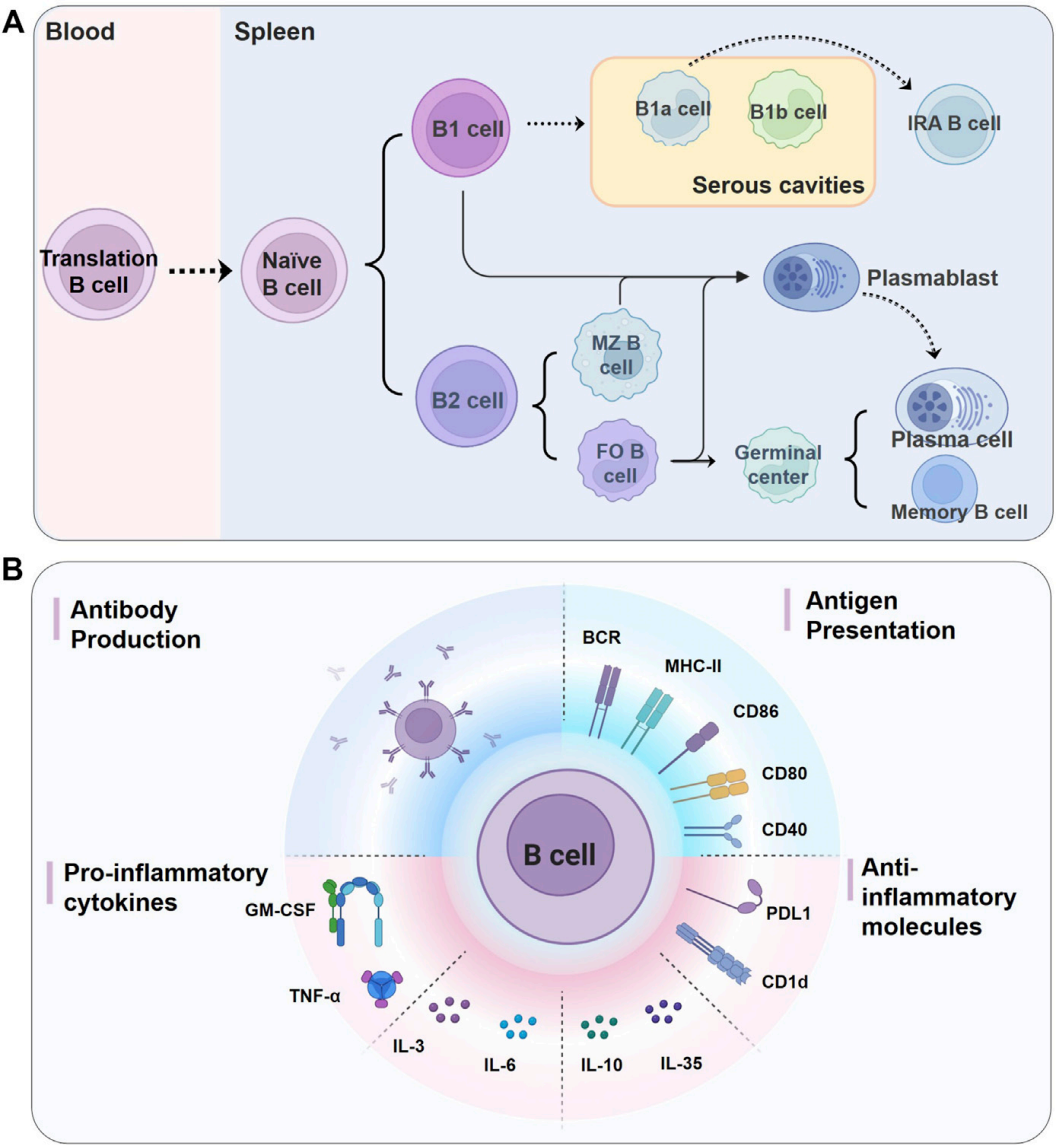
Biology of B cells. **(A)** Different B cell subsets in murine. Naïve B cells functionally mature in the spleen from the immature/transitional cell stages, and can be divided into two lineages, B1 cells and B2 cells. Mature B1 cells migrate into serous cavities and acquire the B1a and B1b cell phenotypes, and B2 cells in the spleen form FO B and MZ B cells. FO B cells make up the majority of mature B cells and are found in the follicles of lymphoid organs, while MZB cells reside in the splenic marginal zone. On encountering antigens, mature B cells either give rise to short-lived plasmablasts or enter the germinal center reaction and eventually differentiate into long-lived plasma cells and memory B cells. Serosal B1a cells can also migrate to the spleen and become IRA B cells, which are characterized by GM-CSF secretion. It is not clear whether plasma cells come from plasmablasts or the early plasmacytosis stage (Nutt et al., 2015). **(B)** B cells perform critical functions in both innate and adaptive immune responses by producing antibodies, presenting antigens to T cells, and producing pro- and anti-inflammatory cytokines and molecules. BCR, B cell receptor; CD, cluster of differentiation; FO, follicular; GM-CSF, granulocyte macrophage-colony stimulating factor; IL: interleukin; IRA: innate response activator; MHC-II, major histocompatibility complex class II; MZ, marginal zone; PDL1, programmed cell death 1 ligand 1; TNF-α, tumor necrosis factor-α.

B cell development begins in the fetal liver and continues in the bone marrow after birth, where bone marrow hematopoietic stem cells (HSCs) differentiate into immature B cells through a series of well-defined stages (Montecino-Rodriguez and Dorshkind, 2012; McLean and Mandal, 2020). When an immature B cell begins to migrate through the bloodstream to the periphery, it first becomes a transitional B cell and then functionally matures into a naïve B cell in secondary lymphoid tissues (Wang et al., 2020a) Although B cells are known for their ability to produce antibodies in the context of adaptive immune responses, they exhibit functional diversity, with various subsets possessing distinct effector roles (Figure 1).

### 2.1 B cell subsets

Naïve B cells are generally divided into two main populations, namely B1 and conventional B2 cells (Figure 1A) (Wang et al., 2020a). B1 cells, which in mice can be further subdivided into B1a and B1b subsets, preferentially accumulate in pleural and peritoneal cavities (Wang et al., 2020a). B1a cells can also transform into granulocyte macrophage-colony stimulating factor (GM-CSF)-producing innate response activator (IRA) B cells upon Toll-like receptors (TLRs) stimulation (Chousterman and Swirski, 2015). However, there is still controversy as to whether existing human B1 cells are functionally equivalent to murine B1 cells (Baumgarth, 2017; Sage et al., 2019). B2 cells encompass a predominant population of follicular (FO) B cells and a smaller population of marginal zone (MZ) B cells (Allman and Pillai, 2008). FO B cells are found mostly in the primary follicles of lymphoid organs and can also recirculate through the blood and lymphatic vessels (Wang et al., 2020a). In mice, MZ B cells reside in the splenic marginal zone and do not leave the spleen like FO B cells; however, in humans, MZ B cells have been observed in the spleen and other secondary lymphoid organs (Doyon-Laliberté et al., 2022). Upon activation by specific antigens, MZ B cells, B1 cells, and FO B cells could differentiate into plasmablasts or short-lived plasma in extrafollicular foci; whereas FO B cells can also form germinal centers (GCs) in follicles and ultimately differentiate into long-lived plasma cells and memory B cells (Adamo et al., 2020). A growing body of evidence supports considering B1 cells as a part of the innate immune system, whereas B2 cells function primarily in adaptive immune responses (Romero-Ramírez et al., 2019).

### 2.2 B cell functions

#### 2.2.1 Antibody production

Arguably, the most studied function of B cells involved in the immune response is antibody production (Figure 1B). Antibodies are secreted by antibody-secreting cells, namely proliferating plasmablasts and non-proliferating plasma cells. And there are two types of plasma cells, including short- and long-lived plasma cells (Nutt et al., 2015). When B cells are activated by antigens, they can differentiate into plasmablasts or short-lived plasma, which is known as “extrafollicular response”; the resulting antibodies tend to have a moderate and unchanged affinity for the antigen, but they are the source of the major early protective antibodies (MacLennan et al., 2003). Activated FO B cells could also proliferate vigorously within primary or secondary lymphoid organs to generate GCs under the influence of specialized T follicular helper (Tfh) cells, where somatic hypermutation, affinity maturation, and class-switch recombination occur. The GCs eventually produce high-affinity, long-lived plasma cells (capable of sustaining a high level of antibody secretion) and memory B cells (Nutt et al., 2015). Unlike FO B cells, B1 and MZ B cells can also spontaneously secrete natural antibody immunoglobulin (Ig) M, even in the absence of exogenous antigens (Savage and Baumgarth, 2015).

#### 2.2.2 Antigen presentation

B cells are also antigen-presenting cells (APCs) and mostly present antigens to activate CD4^+^ T cells to initiate immune responses (Hua and Hou, 2020). After interacting with cognate antigens, B cells internalize and process native antigens and present degraded peptide fragments to antigen-specific CD4^+^ T cells in the context of major histocompatibility complex (MHC) class II molecules (Hua and Hou, 2020). Although B cells are generally considered less efficient as APCs than other professional APCs, such as dendritic cells (DCs), memory B cells are highly efficient in antigen presentation when antigens are presented to T cells that recognize the same antigens (Sabatino et al., 2019). Recent studies further suggested that B cells can act as professional APCs, with the synergistic activation of the B cell receptor (BCR) and the TLRs in response to pathogens or virus-like particles to turn naive T cells into Tfh cells (Hua and Hou, 2020). In this context, B cells are not only potent but are also the predominant APCs. Notably, B cells can also act as APCs to tolerate T cells, such as under conditions of non-BCR-mediated antigen uptake (Oleinika et al., 2019).

#### 2.2.3 Cytokine/chemokine production

B cells can secrete an array of cytokines and chemokines in response to various signals including TLRs activation and BCR engagement, and to regulate other immune cell functions (Adamo et al., 2020). B cells can promote immune responses by secreting various pro-inflammatory cytokines, such as interleukin (IL) -2, IL-3, IL-4, IL-6, and IL-17, interferon-γ (IFN-γ), tumor necrosis factor-α (TNF-α), and GM-CSF, which activate myeloid cells and polarize T-cell responses to drive T helper 1 (Th1) cells and Th17 cells responses (Matsushita, 2019; Oleinika et al., 2019). In contrast, some B cell subsets are able to attenuate inflammation and contribute to immune tolerance through the production of anti-inflammatory mediators, such as IL-10, IL-35, transforming growth factor-β (TGF-β), and through the surface expression of CD1d and programmed cell death 1 ligand 1 (PDL1) (Catalán et al., 2021). These B cells act as negative regulators of immune responses and are often referred to as regulatory B (Breg) cells, which can suppress T cell, monocyte, and DC responses and promote regulatory T (Treg) cell responses (Matsushita, 2019; Boldison et al., 2020; Catalán et al., 2021). An increasing number of studies have shown that imbalances in the number or function of pro-inflammatory B cells and Breg cells exacerbate the dysregulation of immune responses and are involved in the progression of a variety of diseases (Li et al., 2019; Hetta et al., 2020; Busse et al., 2021).

## 3 B cells during sepsis

### 3.1 Numerical, phenotypic, and functional changes of B cells in human sepsis

#### 3.1.1 Adult patients

**FIGURE 2:**
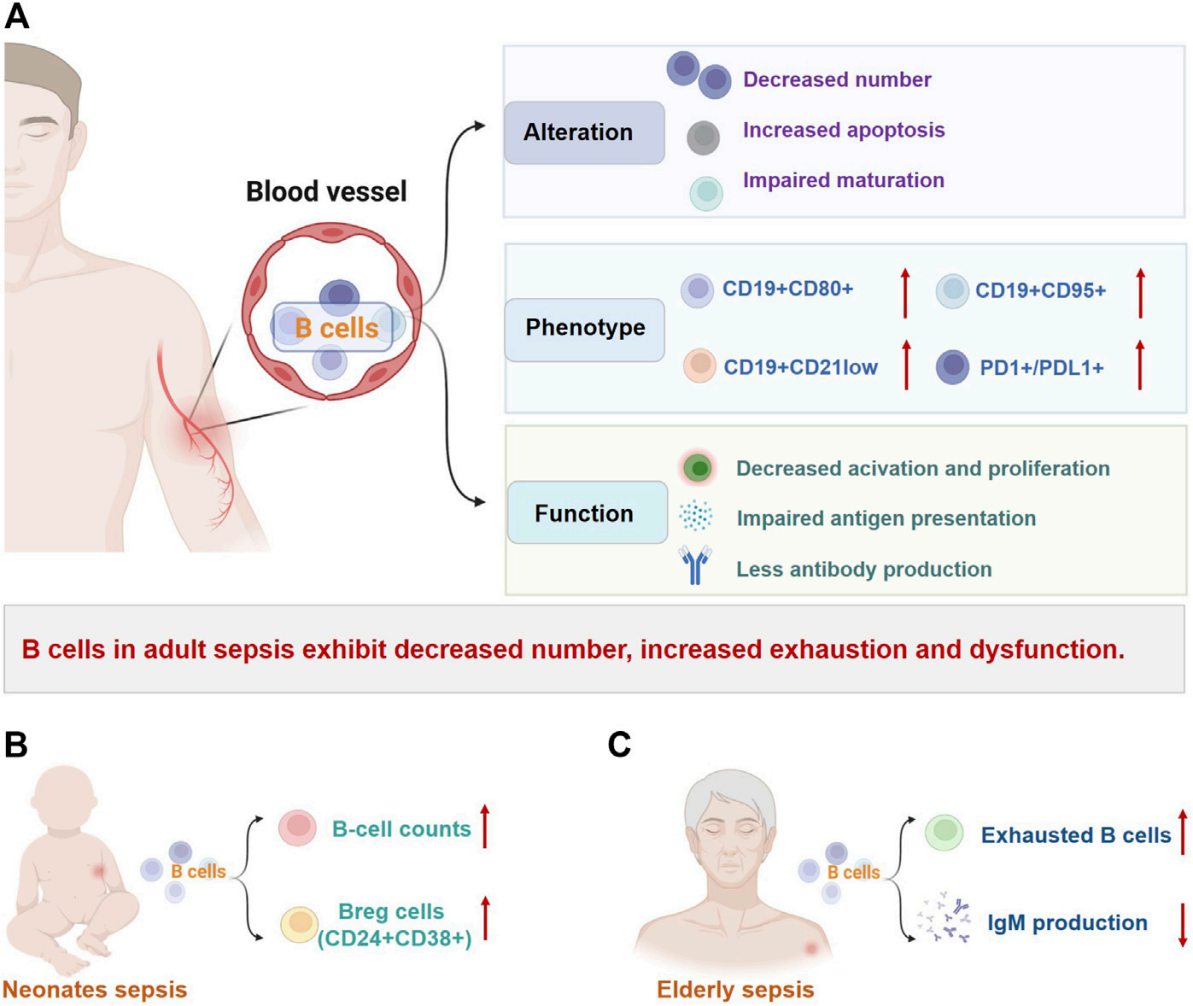
Changes of B cells in sepsis. B-cell number, phenotype, and effector functions are markedly altered in patients with sepsis and are not consistent across different populations. **(A)** In adult patients with sepsis, reduced B-cell number, heterogeneous redistribution, and selective depletion of B-cell subsets are common, along with increased depletion-like/immunomodulatory profiles and decreased immunocompetent B cells. These changes eventually lead to impaired B-cell immune responses in sepsis. **(B)** Unlike in adults, B-cell count and percentage of CD24^+^CD38^+^ Breg cells were significantly increased in neonatal sepsis. **(C)** Elderly patients with sepsis exhibit increased exhausted B cells and dysfunction such as decreased immunoglobulin production. Abbreviations: Breg: regulatory B cell; CD, cluster of differentiation; Ig, immunoglobulin; PD1, programmed cell death 1; PDL1, programmed cell death 1 ligand 1.

There is substantial evidence that the number of circulating B lymphocytes in adults with sepsis is significantly reduced, which is associated with poor prognosis (Figure 2A; Table 1) (Hotchkiss et al., 2001; Holub et al., 2003; Gogos et al., 2010; Venet et al., 2010; Andaluz-Ojeda et al., 2011; Monserrat et al., 2013; Suzuki et al., 2016; Shankar-Hari et al., 2017a; Gustave et al., 2018; Wilson et al., 2018; Brinkhoff et al., 2019; Dong et al., 2020; Duan et al., 2020). This lymphopenia affects the changes of B-cell phenotype heterogeneously, with a marked reduction of CD19^+^CD23^+^ (activated regulatory B cells) and CD19^+^CD5^+^ B cells (natural responder B1a cells), but normal numbers of CD19^+^CD69^+^ B cells (early activated B cells) (Monserrat et al., 2013). The percentages of CD19^+^ B cells expressing CD95^+^ (a marker of cell surface death receptor, also known as Fas) and CD80^+^ (a functionally critical antigen in cells activation) were significantly increased in non-surviving patients, which indicated that the B cells apoptosis is detrimental to host survival (Monserrat et al., 2013). Subsequent studies have found that although the number of B cells decreases, the proportion of B cells in total lymphocytes seems to increase, while the remaining circulating B cells show an increased proportion of CD21^low^CD95^hi^ B cells (exhausted-like B cells) (Suzuki et al., 2016; Gustave et al., 2018). Dong et al. explained that the increased CD21^low^ cells were tissue-like memory and activated memory B cells (Dong et al., 2020).

**TABLE 1:** The changes of B cells in adult patients with sepsis.

Year/Authors	Subjects	Testing time	Main findings	References
Hotchkiss et al. (2001)	27 sepsis patients 8, non-sepsis patients	postmortem	Splenic B cells were reduced in patients with sepsis, and the loss of B cells was more severe with a prolonged course of sepsis	Hotchkiss et al. (2001)
Holub et al. (2003)	32 sepsis patients 34 healthy controls	Day 1, 3, 7, and 14 after admission	B-cell counts were not changed in sepsis patients, but decreased B-cell counts on days 3 and 7 were associated with nosocomial infections	Holub et al. (2003)
Gogos et al. (2010)	505 sepsis patients	Within 24 h after diagnosis	Absolute B-cell counts were lower in patients with severe sepsis/shock compared to sepsis; B-cell counts within 24 h of diagnosis in patients correlate with infectious pathogens	Gogos et al. (2010)
Venet et al. (2010)	21 septic shock patients	Within 2 h after diagnosis and the next 48 h	B-cell numbers were reduced within 48 h	Venet et al. (2010)
Andaluz-Ojeda et al. (2011)	50 patients with severe sepsis or septic shock	Day 1, 3, and 10 after admission	Non-survivors had decreased B cell numbers on both days 3 and 7, but not significantly compared with survivors	Andaluz-Ojeda et al. (2011)
Monserrat et al. (2013)	52 septic shock patients 36 healthy controls	On the day of admission and 3, 7, 14, and 28 days later	The counts of CD23^+^ B cells and CD5^+^ B cells were reduced, while the proportion of CD23^+^ B cells and CD69^+^ B cells is increased at ICU admission. The absolute counts of CD40^+^ B cells were decreased and the percentages of CD95^+^ and CD80^+^ B cells were increased in non-surviving patients	Monserrat et al. (2013)
Suzuki et al. (2016)	33 severe sepsis patients, 44 healthy controls	Within 72 h after diagnosis and at 8–11 days later	Naive B-cell populations were reduced in chronic sepsis patients compared with HCs and acute sepsis patients; the percentage of CD21-/low B cells was increased in sepsis patients	Suzuki et al. (2016)
Shankar-Hari et al. (2017a)	101 sepsis patients	On the day of admission	The proportions of plasmablasts, IgM memory B cells, and class-switched memory B cells were reduced; B-cell apoptosis was highest in the memory subsets	Shankar-Hari et al. (2017a)
Gustave et al. (2018)	138 septic shock patients 48 healthy controls	Day 1, 2, and 6 after diagnosis	B-cell counts decreased but the percentage increased; a strong decrease in HLA-DR expression appeared on day 1 and persisted through day 6; the proportion of CD21low CD95high B cells was increased	Gustave et al. (2018)
Wilson et al. (2018)	22 sepsis patients 11 healthy controls	Within 12 h of ICU admission	Higher percentages of PD-1 and PD-L1 in CD27^+^ and CD27^−^ B cells and higher percentages of PD-L2 in CD27^+^ B cells in sepsis	Wilson et al. (2018)
Brinkhoff et al. (2019)	20 healthy volunteers (LPS or placebo injection)	Up to 72 h after injection	At 3 h after LPS injection, the absolute numbers of B cells decreased, while the relative proportions of naive B cells and plasmablasts increased, and the percentage of memory B cells decreased	Brinkhoff et al. (2019)
Dong et al. (2020)	33 septic shock patients 10 healthy controls	Day 1, 3, and 7	Patients with septic shock had lower immature transitional (IM) B cells and resting memory (RM) B cell counts, higher percentages of tissue-like memory B and activated memory B cells, and lower percentages of IM B and RM B cells	Dong et al. (2020)
Duan et al. (2020)	40 sepsis patients	Within 24 h of the onset and 24 h later	Non-survivors had lower numbers of B and Tfh cells, and the main difference in subpopulations was not naive B cells, but mature B cells	Duan et al. (2020)

The heterogeneous redistribution and selective depletion of B-cell subsets in sepsis have also been observed. There are no differences between sepsis patients and healthy controls in the frequency of transitional B cells and naïve cells, whereas the proportion of memory B-cell loss was high (Shankar-Hari et al., 2017a). This alteration is also present across different sepsis outcomes, as non-survivors have lower numbers of mature B cells (Duan et al., 2020). Several studies have suggested that plasma cells as well as IL-10-producing Breg cells increase significantly in patients with sepsis and return to normal upon recovery (Harada et al., 1996; Gustave et al., 2018; Li et al., 2018). Indeed, the increased plasma cells may possess an immunoregulatory profile, as recent studies have found that sepsis expands the population of IL-10-producing plasmablasts that can exacerbate immunosuppression (Shen et al., 2014).

Circulating B cells in patients with sepsis exhibit decreased expression of co-stimulatory molecules, such as MHC-II and CD40, which reflects that the antigen-presentation capacity of circulating B cells may be strongly altered (Ditschkowski et al., 1999; Gustave et al., 2018; Schenz et al., 2020). Meanwhile, the expression of inhibitory immune checkpoint molecules, PD-1/PD-L1, is increased in B cells (Wilson et al., 2018). Besides, B-cell function is markedly altered after sepsis, with reduced ability to activate and proliferate and decreased ability to secrete antibodies because of insufficient IgM and IgG synthesis (Giamarellos-Bourboulis et al., 2013; Suzuki et al., 2016; Dong et al., 2020). As a matter of fact, hypoimmunoglobulinemia is common in patients with sepsis, although the exact relationship with mortality is not well established (Akatsuka et al., 2021; Alagna et al., 2021).

#### 3.1.2 Children and elderly patients

Alterations in B-cell populations have also been observed in sepsis in children (Figure 2B) and the elderly (Figure 2C), although there are only a few studies (Table 2). Contrary to observations in adults, neonates with sepsis and suspected infection have significantly increased B-cell counts and percentages on the day of sepsis diagnosis and 7 days later, with a marked decrease in naïve B cells, an increase in transitional B cells, and persistently high levels of plasma cells (Hotoura et al., 2011; Hotoura et al., 2012; Li et al., 2018). The percentage of IL-10-producing CD19^+^CD24^hi^CD38^hi^ Breg cells in neonatal sepsis with good prognosis is also significantly increased compared with healthy controls (Pan et al., 2016; Li et al., 2018). Interestingly, in children with septic shock (ages from 1 month to 18 years), although B cell counts are reduced, they are only modestly affected compared to the marked reduction in total lymphocyte counts (Remy et al., 2018). In elderly patients with sepsis, reduced immunocompetent B cells with increased CD21^-/low^ exhausted B cells and insufficient IgM production can be observed, which may increase susceptibility to secondary infections (Suzuki et al., 2016). Another study showed that peripheral blood CD20^+^CD24^hi^CD38^hi^ Breg cells levels were significantly elevated in survivors compared with non-survivors in elderly patients with sepsis (Wang et al., 2017). Obviously, the changes of B cells in sepsis are not the same among different age groups, which may be related to the immune system characteristics of patients. Considering the limited number of studies on sepsis in children and the elderly, further research is needed.

**TABLE 2:** The changes of B cells in Children and elderly patients with sepsis.

Year/Authors	Subjects	Testing time	Main findings	References
Hotoura et al. (2011)	Full-term neonates; 25 sepsis patients 20, suspected infection 50, controls	Before treatment, 2 days after treatment, 48 h after treatment stops	The percentage of B cells in sepsis group and suspected group was higher than that in control group before and 2 days after treatment; the percentage of B cells in sepsis group remained unchanged before and after treatment	Hotoura et al. (2011)
Hotoura et al. (2012)	Preterm neonates; 17 sepsis patients 25, suspected infection 40, controls	Before treatment, 2 days after treatment, 48 h after treatment stops	Before treatment, the percentage of B cells in sepsis group was higher than that in control group; the percentage of B cells in sepsis group after 48 h of treatment was lower than that before treatment	Hotoura et al. (2012)
Pan et al. (2016)	30 neonatal sepsis, numbers of controls: not provided	Not provided	The percentage of CD19^+^CD24hiCD38hi Breg cells and IL-10-producing Breg cells in sepsis patients was significantly higher than that in controls	Pan et al. (2016)
Duan et al. (2020)	16 neonatal sepsis 20, controls	Day 7, 14, and 21 after diagnosis	The number and frequency of B cells increased on day 7 and 14, and returned to normal on day 21, among which IL-10-producing transitional B cells are the main elevating B cell subset.	Duan et al. (2020)
Remy et al. (2018)	26 pediatric septic shock, 30 controls	Day 1–2, 3–5, and 7–9 after the onset	The absolute B-cell count in children with sepsis decreased slightly	Remy et al. (2018)
Wang et al. (2017)	58 elderly sepsis	Day 1, 3, and 7 after diagnosis	The percentage of CD19^+^CD24hiCD38hi Breg in the death group was lower than that in the survivor group	Wang et al. (2017)

### 3.2 Numerical, phenotypic, and functional changes of B cells in animal models

A prominent reduction in B-cell numbers and percentage has also been observed in septic mice (Mohr et al., 2012; Kulkarni et al., 2018; Sjaastad et al., 2018; Tao et al., 2019; Taylor et al., 2020a; Umakoshi et al., 2020; Vu et al., 2022) (Table 3). The B-cell numbers in the blood, spleen and inguinal lymph nodes were significantly reduced 2 days after sepsis induction, decreasing by 11-fold, 2-fold, and 6-fold, respectively, before recovering to sham levels by day 30 (Sjaastad et al., 2018). The frequency of B cells in peripheral blood decreased at 6 h and was still markedly decreased at 7 days (Umakoshi et al., 2020). In contrast, the decrease in T-cell number was not apparent at 6 h, indicating that B lymphocytopenia was more evident and was one of the earliest events in the cecal ligation and puncture (CLP)-induced sepsis (Umakoshi et al., 2020). What’s more, septic mice exhibited differential depletion of B-cell subsets. Splenic FO, MZ, and GC B-cell counts were significantly reduced in CLP-induced sepsis mice, whereas plasma cells were markedly increased at 72 h (Taylor et al., 2020a). However, it is worth noting that the changes in B-cell subsets may vary in animal models of sepsis induced by different treatments, which may be related to the complex pathogenesis of sepsis, as detailed in Table 3. For example, the frequency of CD1d^+^/CD5^+^ Breg cells in the peripheral blood of CLP mice increased at 6 h and returned to baseline at 7 days (Umakoshi et al., 2020). In septic mice induced by intraperitoneal injection of fecal suspension, the percentage and number of Il-10 + Breg cells were 3-fold higher at 1 month after treatment than controls (Kulkarni et al., 2018). Diversely, in mice with sepsis induced by intraperitoneal injection of LPS, the decreased CD5^+^CD1dhi Bregs and IL-10-producing B cells in severe endotoxic shock mice were observed compared with controls (Tao et al., 2019). Additionally, the frequency and number of B1 cells in the peritoneal cavity of mice were prominently reduced after CLP surgery, which may be due to the relocation of B1 cells to other organs (Kelly-Scumpia et al., 2011).

**TABLE 3:** The changes of B cells in animal models of sepsis.

Year/Authors	Models	Tissues	Main findings	References
Kelly-Scumpia et al. (2011)	CLP	Peritoneal, secondary lymphoid organs, bone marrow	Peritoneal B1 cells decreased within 36 h after sepsis and migrated to spleen and lymph nodes; an increase in CD69+B cells in the spleen, bone marrow, and lymph nodes as early as 24 h after CLP.	Kelly-Scumpia et al. (2011)
Mohr et al. (2012)	CLP	Spleen	The number of splenic B cells reduced and the proportion of CD86^+^, MHC II + splenic B cells increased on day 2 after CLP.	Mohr et al. (2012)
Sjaastad et al. (2018)	CLP	Blood, secondary lymphoid organs	The number of B cells in the blood, spleen, and inguinal lymph nodes significantly reduced 2 days after CLP, with 11-fold, 2-fold, and 6-fold, respectively, and recovering to sham levels by day 30	Sjaastad et al. (2018)
Kulkarni et al. (2018)	Intraperitoneal injection of fecal suspension	Spleen	The percentage and number of IL-10 + Breg cells were 3-fold higher than control group after 1 month, but no longer increased after 3.5 months of sepsis	Kulkarni et al. (2018)
Tao et al. (2019)	Intraperitoneal injection of LPS	Spleen	Reduced CD5^+^CD1dhi Bregs and IL-10 production, and increased FasL + B cells in severe endotoxic shock mice; the gene level of proinflammatory, anti-inflammatory, and B cell-related markers were markedly altered	Tao et al. (2019)
Umakoshi et al. (2020)	CLP	Blood	The frequency of B cells decreased at 6 h and continued for 7 days; the frequency of CD5^+^CD1d+ Breg cells increased at 6 h and returned to baseline at day 7	Umakoshi et al. (2020)
Taylor et al. (2020a)	CLP	Spleen	Reduced total splenic B cells, splenic FO, MZ, and GC B cells decreased, whereas increased plasma cells at 72 h	Taylor et al. (2020a)
Vu et al. (2022)	CLP mice with secondary *Candida* infection	Spleen	Decreased splenic B cell numbers and increased proportions of PD1+B cells until day 12 post-CLP.	Vu et al. (2022)

In concert with the findings in human sepsis, the inhibitory receptors, PD-1 and PD-L1, as well as the apoptosis receptor, CD95, were significantly elevated on B cells in response to CLP-induced sepsis (Tao et al., 2019; Umakoshi et al., 2020; Vu et al., 2022). Interestingly, an improved state of B-cell activation was seen in septic mice by the higher proportion of CD69^+^, CD86^+^, and MHC-II expressing splenic B cells early after CLP (Kelly-Scumpia et al., 2011; Mohr et al., 2012). Transcriptome analysis showed that the gene expression levels of various proinflammatory cytokines, such as IL-3, IL-6, IFN-γ, and TNF-α, were elevated, whereas those of the anti-inflammatory cytokines IL-10 and TGF-β1 were decreased in septic mice (Tao et al., 2019). Also, B-cell activation-related genes such as CD25, CD40, CD80, and CD86 were upregulated, while genes of some surface markers associated with Breg cells, including CD5, CD1d, and CD21, were downregulated (Tao et al., 2019). These results further suggest that B cells have increased apoptosis and altered function in sepsis.

### 3.3 Potential mechanisms of B-cell depletion in sepsis

The mechanisms leading to the profound depletion of B cells in adult patients with sepsis and septic mice are not well-understood; however, several potential explanations have been proposed. In theory, impaired bone marrow production could be the direct cause of reduced B-cell numbers; however, no differences in the number of naïve B cells and cytokines that inhibited and promoted B-cell proliferation were observed between survivors and non-survivor (Duan et al., 2020). These findings indicate that reduced B-cell numbers are not a consequence of a bone marrow production disorder. Accelerated B-cell apoptosis is considered a reason for the decrease in B-cell numbers. Previous evidence has revealed that extensive B-cell apoptosis occurs in blood, intestines, and peripheral lymphoid organs and causes progressive profound loss of B lymphocytes in sepsis patients and experimental animals (Hotchkiss et al., 1997; Ayala et al., 1998; Hotchkiss et al., 1999; Hotchkiss et al., 2005). Indeed, compared with naïve B cells, memory B cells are more susceptible to sepsis-induced activation-associated cell death (Chang et al., 2016). Studies have shown that there may be multiple triggers for B-cell apoptosis, including activation of extrinsic cell surface death receptor (Fas)/Fas ligand (FasL)- and intrinsic mitochondria-mediated pathways (Ayala et al., 1998; Chung et al., 1998; Chung et al., 2001; Hotchkiss et al., 2005). Besides, impaired B-cell maturation may also account for their reduced numbers. It has been demonstrated that secondary lymphoid organs taken from septic patients have a lower cellular density compared with healthy controls and that a reduction in the number of circulating Tfh cells correlates with the decreased numbers of B cells (Hotchkiss et al., 2001; Duan et al., 2020). These results suggest that the loss of APCs and Tfh cells may impair B-cell maturation and ultimately lead to a reduction in B-cell number (Boomer et al., 2011; Sjaastad et al., 2018). Moreover, animal models have indicated that B cells exposed to B-cell activating factor (BAFF) and lipopolysaccharide (LPS) were more susceptible to Fas-mediated cell death than B cells cultured in absence of BAFF (Acosta-Rodriguez et al., 2007). Similarly, human endotoxemia models have revealed that the dramatic increase in BAFF serum levels after the LPS challenge was associated with memory B cell depletion (Brinkhoff et al., 2019). These results suggest that BAFF may be another factor contributing to B-cell deficiency in sepsis, although it promotes B-cell maturation under physiological circumstances (Brinkhoff et al., 2019). In addition, the redistribution of circulating B cells into peripheral tissues under certain circumstances is an alternative explanation for the drop in B-cell numbers and the skewing of the B-cell compartment (Engler et al., 2004).

## 4 The key role of B cells in sepsis

**FIGURE 3:**
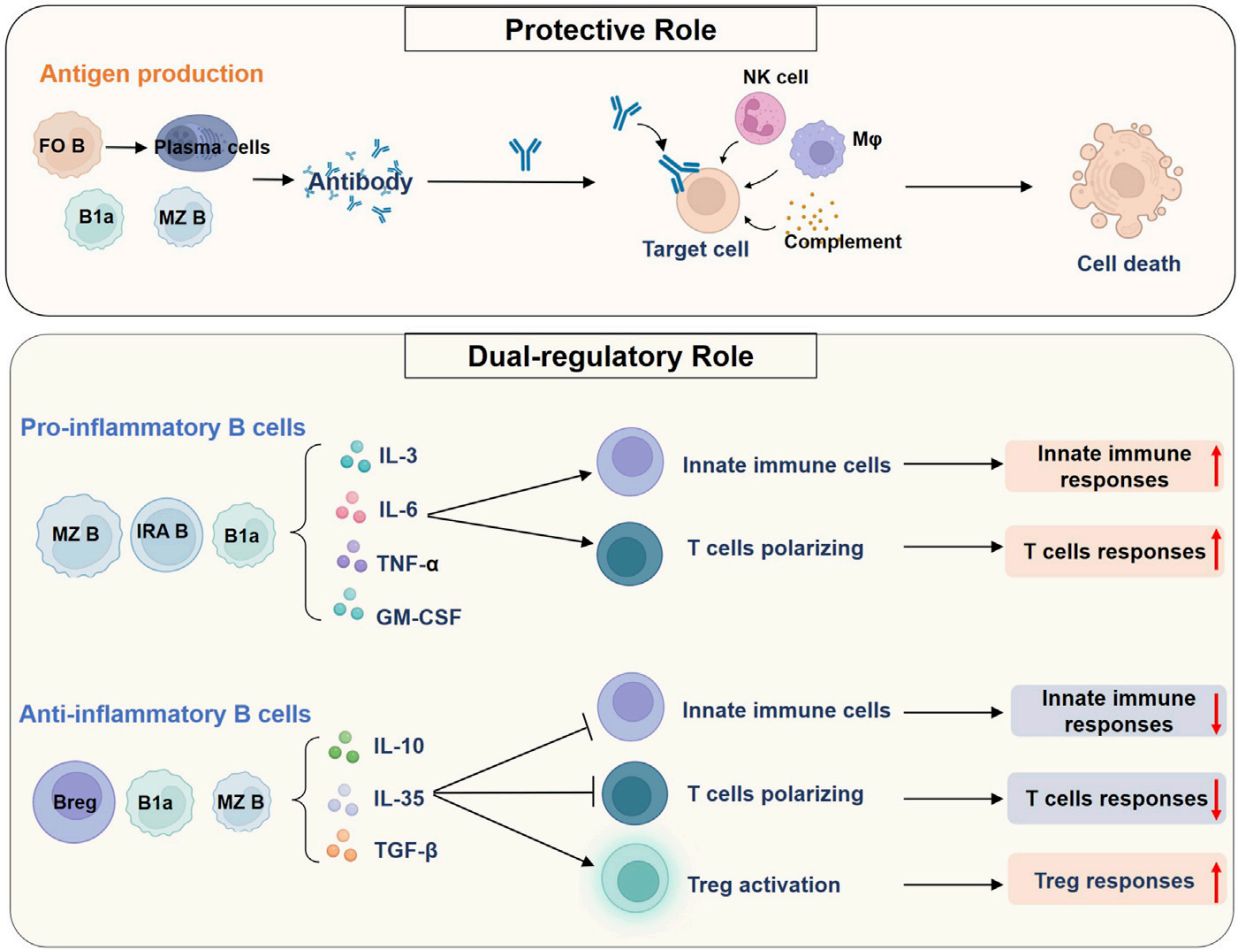
Roles of B cells in sepsis. B cells can perform a protective role in pathogen clearance and reduction of endotoxemia in sepsis by producing antibodies. FO B cells are the main source of high-affinity antibody-producing cells, whereas B1a cells and MZ B cells could give rise to natural antibodies. Antibodies bind to antigens on cellular targets and could cause complement-dependent cytotoxicity, antibody-dependent cellular phagocytosis, and antibody-dependent cellular cytotoxicity. A variety of leukocytes, such as NK cells and macrophages, and the complement system are involved in the process. B cells could also play a dual regulatory role in sepsis, which is mediated by producing various cytokines. On the one hand, B cells such as MZ B cells, IRA B cells, and B1a cells produce large amounts of pro-inflammatory cytokines during sepsis. These cytokines can modulate the innate immune cells and promote the polarization of CD4^+^ T cells toward multiple phenotypes, thereby enhancing both innate and adaptive immune responses. On the other hand, Bregs can act as negative regulators of immune responses by secreting anti-inflammatory cytokines. In mice, multiple types of IL-10-producing B cells have been found, including MZ B cells and B1a cells. These B cells inhibit the pro-inflammatory activity of innate immune cells and Th cells, and promote Treg responses, ultimately inducing endotoxin tolerance and immunosuppression in sepsis. Abbreviations: Breg, regulatory B cell; FO, follicular; GM-CSF, granulocyte macrophage-colony stimulating factor; IL, interleukin; IRA, innate response activator; MZ, marginal zone; Mφ, macrophage; NK cell, natural killer cell; TGF-β, transforming growth factor-β; TNF-α, tumor necrosis factor-α; Treg, regulatory T cell.

B cells are involved in the host immune response to sepsis as immunomodulators. On the one hand, B cells can perform a protective role in pathogen clearance and reduction of endotoxemia in sepsis by producing antibodies; on the other hand, B cells play a dual regulatory role in sepsis by secreting cytokines and chemokines and affecting other leukocyte functions (Figure 3).

Antibody production is the most widely recognized role of B cells. Antibodies bind to their target antigens and promote Fc receptor-mediated killing of target cells by leukocytes such as natural killer cells, macrophages, and neutrophils. This process is accomplished by the phagocytosis or release of cytotoxic particles, termed antibody-dependent cellular phagocytosis (ADCP) and antibody-dependent cytotoxicity (ADCC), respectively (Lu et al., 2018). The classical complement pathway can also be activated by IgG and IgM antibody isotypes, causing complement-dependent cytotoxicity (CDC) (Lu et al., 2018). Besides, some B-cell subsets, mainly B1a cells, can also spontaneously secrete natural antibodies to neutralize endotoxins and clear circulating pathogens in the absence of external antigenic stimulation (Reid et al., 1997).

B cells can produce a large number of pro-inflammatory cytokines such as IL-3, IL-6, GM-CSF, and TNF-α to enhance the systemic inflammatory responses in sepsis (Rauch et al., 2012; Weber et al., 2015; Honda et al., 2016). B cell-mediated cytokine secretion also plays a key role in polarizing CD4^+^ T cells responses to drive Th1 cells and Th17 cells responses and in activating innate immune cells such as DC and monocytes to defend against infection (Tsay and Zouali, 2018; Lo et al., 2021; Podstawka et al., 2021). Meanwhile, Breg cells can also negatively regulate the immune responses, mainly by producing anti-inflammatory factors such as IL-10 and IL-35 to induce endotoxin tolerance or immunosuppression, inhibit innate immune responses and promote Treg responses in sepsis (Barbeiro et al., 2011; Tao et al., 2019; Dasgupta et al., 2020). Therefore, it can be concluded that the balance of pro-inflammatory B cells and Breg cells is critical for improving immune dysfunction in sepsis.

Unfortunately, the role of B cells in sepsis has been relatively poorly studied compared to that of T cells, and most of the data collected so far have come from animal models of sepsis. Herein, we summarized the roles of the preponderance B-cell subsets to provide a framework for understanding the roles of B cells in sepsis.

### 4.1 Follicular B cells

FO B cells function as the major component of the T cell-dependent B cell adaptive immune response and produce high affinity antibody-producing plasma cells (Wang et al., 2020a). Plasma cells produce antibodies that bind to antigens on cellular targets, resulting in ADCC, ADCP, or CDC (Lu et al., 2018). During sepsis, FO B cells can be activated through type I interferon (IFN-I) receptor, and IFN-I-activated FO B cells have a protective early innate immune response role during bacterial sepsis (Kelly-Scumpia et al., 2011). CLP markedly altered FO B cells responses, resulting in severely impaired antigen-specific primary antibody production that may persist for at least 30 days (Mohr et al., 2012; Sjaastad et al., 2018). The prolonged impairment in primary CD4^+^T cell-dependent FO B cells responses is partly due to the insufficient help from Tfh cells in GC (Sjaastad et al., 2018). Taylor et al. further discovered the contribution of pre-existing T cell memory in mice *via* the administration of an anti-CD3 activating antibody, a procedure called “immune education” (Taylor et al., 2020b). They found that CLP reduced the number of FO B cells in control mice, but in educated mice, no such change was noticed 24 h after CLP (Taylor et al., 2020a). Importantly, immune education induced prior to CLP not only contributes to the persistent general changes to B-cell response 60 days after CLP but also increases antigen-specific FO B cells responses (Taylor et al., 2020a). These findings demonstrated that pre-existing Tfh cells assist in preventing the depletion of FO B cells and in rescuing the FO B cells in response to CLP. Besides, the heterogeneity of Tfh cells underscores their ability to modulate distinct humoral immune responses to different pathogens, all of which have distinct consequences (Olatunde et al., 2021). In addition to Tfh cells, follicular DCs are also involved in the functional regulation of FO B cells. A recent study has shown that the impairment of antigen-specific FO B cell humoral responses in CLP mice may be due to follicular DCs dysfunction rather than due to primarily intrinsic defects in the B-cell compartment (Rana et al., 2021).

### 4.2 Marginal zone B cells

Splenic MZ B cells play a critical role in rapidly induced production of natural antibodies or T-cell-dependent and -independent responses against blood-borne foreign antigens, and the dysfunction of MZ B cells could result in an attenuated adaptive humoral response (Liu et al., 2021). In sepsis, MZB cells are the front-line defenders of humoral immunity because MZ B cells are highly sensitive to bacterial components such as LPS and are ready to produce antibodies rapidly and stably (Gunn and Brewer, 2006). Mice deficient in MZ B cells showed decreased antibody secretion in the early phase after pathogen invasion into the blood circulation (Guinamard et al., 2000). MZ B cells are also involved in inflammatory responses by secreting various cytokines, such as IL-6 and IL-10, and by regulating the functions of other immune cells (Bialecki et al., 2009). Indeed, MZ B cells play a dual regulatory role in the inflammatory response to sepsis. On the one hand, MZ B cells can perform a pro-inflammatory role in sepsis (Shibuya et al., 2017). In a mouse model of sepsis induced by intravenous injection of LPS or *Escherichia coli (E.coli)*, MZ B cells could produce large amounts of inflammatory cytokines and chemokines *via* Fcα/μR-coupled Toll-like receptor four pathways, such as IL-6, and exacerbate systemic inflammatory responses and endotoxic shock in mice (Honda et al., 2016)*.* Mice deficient in MZ B cells and IL-6 have attenuated systemic inflammatory responses and prolonged survival compared with wild-type mice (Honda et al., 2016). Additionally, MZ B cells secrete IL-6 and CXCL1/CXCL2 in the early 3–4 h after *Staphylococcus aureus (S. aureus)* infection, recruiting neutrophils to the site of infection to rapidly eliminate invading pathogen, and MZ B cell-deficient mice are more susceptible to systemic *S. aureus* infection compared with wildtype mice (Lo et al., 2021). On the other hand, MZ B cells also play a role in suppressing inflammation in infection or sepsis. MZ B cells are the most potent IL-10-producing cells *in vitro*, and increased MZ B cells attenuate early inflammatory responses and primarily exhibited anti-inflammatory effects during the early stage of sepsis in Gpr174-deficient mice (Zhu et al., 2020). Similarly, MZ B cells are also a major source of IL-10 in the context of *Listeria monocytogenes* infection, and MZ B cell-deficient mice showed decreased bacterial load and increased survival (Lee and Kung, 2012). Therefore, MZ B cells can play a dual regulatory role in the inflammatory response to sepsis by producing pro-inflammatory factors such as IL-6 and anti-inflammatory factors such as IL-10.

### 4.3 B1a cells

B1a cells are the main subgroup of B1 cells with profound immunomodulatory functions (Wang et al., 2020a). B1a cells are capable of spontaneously secreting steady-state levels of natural IgM as well as of responding to infections with induced IgM production, which can defend against infection by different pathogens (Ordoñez et al., 2018; Smith and Baumgarth, 2019). Besides, B1a cells can produce GM-CSF, which governs emergency myelopoiesis, and anti-inflammatory cytokine IL-10 spontaneously or following infection, which reduces inflammation and tissue damage (Aziz et al., 2015; Smith and Baumgarth, 2019). Conversely, B1a cells also produce IL-3, IL-6, IL-17, and TNF-α, which play proinflammatory roles in sepsis (Aziz et al., 2015; Smith and Baumgarth, 2019). Substantial evidence suggests that B1a cells play a protective role in sepsis and provide a survival benefit. Natural IgM and IgG3 secreted by B-1a cells can function to neutralize endotoxins and play an important role in the clearance of pathogenic substances from the circulation (Reid et al., 1997). It has been speculated that B1a cell-sourced natural IgM could also facilitate the clearance of apoptotic cells by phagocytes, which might aid in the amelioration of sepsis (Aziz et al., 2015). CLP mice deficient in secreted (s) IgM showed a significant increase in mortality as compared with their wild-type counterparts, which was associated with decreased neutrophils infiltration to clear peritoneal bacteria and elevated levels of endotoxin and proinflammatory cytokines in the circulation (Ayala et al., 1998). Conversely, resistance to CLP by sIgM-deficient mice was restored by reconstitution with polyclonal IgM from normal mouse serum (Ayala et al., 1998). These evidences suggest that in sepsis, B1a cells can immediately defend against severe bacterial infection by secreting antibodies. In addition, B1a cells can also secrete cytokines to exert modulatory function. In murine models of LPS-induced sepsis and bacterial sepsis, B-1 cell-deficient mice showed exacerbated sepsis severity and increased mortality, accompanied by increased levels of proinflammatory cytokines TNF-α and IL-6 and decreased levels of IL-10 in the plasma, lung, and gut (Barbeiro et al., 2011; Aziz et al., 2017). Adoptive transfer of B1a cells into septic mice markedly attenuated systemic inflammation and organ damage, and ultimately improved survival, in which IL-10 produced from B1a cells plays a key protective role because the mice treated with IL-10-deficient B1a cells were not protected against sepsis (Aziz et al., 2017; Aziz et al., 2018). It has been further identified that IL-10 production by B1a cells is regulated by cAMP-response element binding protein through its nuclear translocation and binding to putative responsive elements on the IL-10 promoter (Aziz et al., 2017). Of note, recent studies have discovered a unique renin-expressing B1 lymphocyte, which can synthesize renin and trap and phagocytose bacteria and is effective in bacterial killing, suggesting that B1 cells may exert antibacterial properties through multiple mechanisms (Belyea et al., 2021).

### 4.4 Innate response activator B cells

It has been demonstrated that IRA B cells are derived from B1-a cells, that is, peritoneal and serosal B1a cells can migrate to the spleen and lung and transform into IRA B cells, which expresses GM-CSF after LPS stimulation (Chousterman and Swirski, 2015). There is evidence that IRA B cells can be both protective and detrimental in sepsis, depending on the cytokines they produce. IRA-B cells could recognize and clear bacterial infection in innate immunity through pattern recognition receptors and secretion of pro-inflammatory GM-CSF (Rauch et al., 2012). In a murine model of sepsis, mice with a B cell-restricted deficiency in GM-CSF showed increased neutrophil infiltration to the peritoneum; however, these neutrophils had impaired phagocytic activity, and the mice experienced an impaired bacterial clearance and a severe cytokine storm, which precipitated septic shock. Based on these observations, IRA B cells are the gatekeepers of bacterial infection and exert a protective role during sepsis by their provision of GM-CSF (Rauch et al., 2012; Robbins and Swirski, 2012). In contrast to the reported beneficial role of IRA B cells in sepsis, Weber et al. showed that IRA B cells secreted pro-inflammatory IL-3 to potentiate inflammation in sepsis, which might degrade sepsis outcomes (Weber et al., 2015). Using a mouse model of abdominal sepsis, they showed that IL-3 sourced from IRA B cells induced myelopoiesis of Ly-6C^hi^ monocytes and neutrophils and fueled an uncontrolled cytokine storm; in contrast, IL-3 deficiency protected mice against sepsis. More importantly, in human sepsis, IL-3 aggravates disease exacerbation, and high plasma IL-3 levels are associated with high mortality, even after adjusting for prognostic indicators (Weber et al., 2015). Thus, IRA B cells and their IL-3 production exacerbate the adverse consequences of sepsis. Indeed, genes encoding GM-CSF and IL-3 are contiguous in mouse and human chromosomes, and both GM-CSF and IL-3 belong to the β common (βc/CD131) chain family of cytokines, which exhibit pleiotropic functions to leukocyte production, proliferation, and survival (Chousterman and Swirski, 2015; Chin et al., 2019). Taken together, it can be concluded that IRA B cells may lead to diametrically opposite outcomes through producing different proportions of dominant cytokines in sepsis under certain conditions, and a fine-tuned balance diminishing IL-3 production while retaining GM-CSF secretion in IRA B cells is essential for the proper immune response against pathogens and the prevention of excessive damage during sepsis.

### 4.5 Regulatory B cells

The exact source of Breg cells remains elusive, but nearly all B-cell subsets, namely immature B cells, mature B cells, and plasma cells, can differentiate into Breg cells in mice and humans (Rosser and Mauri, 2015). Breg cells are now widely accepted as important regulatory components of the immune system. They provide the host with immune tolerance and immunomodulatory functions, which can be mediated by the production of anti-inflammatory cytokines, such as IL-10, IL-35, and TGF-β, and ensuing suppression of T cells (Wang et al., 2020b; de Mol et al., 2021). It has been shown that B lymphocytopenia with the appearance of Bregs is the earliest event, likely leading to immunoparalysis in sepsis (Umakoshi et al., 2020). The level of CD20^+^CD24^hi^CD38^hi^ Breg cells is elevated in neonatal sepsis and has a more potent inhibitory effect on naïve CD4^+^ T cells; however, it is decreased in non-survival elderly sepsis compared with survivors and is an independent risk factor for the prognosis (Pan et al., 2016; Wang et al., 2017). Tao et al. reported that IL-10 but not FasL expression in CD5^+^CD1d^hi^ Breg cells in response to endotoxin was dramatically reduced in severe septic shock mice, and the regulatory function of Breg cells *in vitro* to control the Th1 response was also diminished. Adoptive transfer of CD5^+^CD1d^hi^ Breg cells from healthy wild-type mice but not that from IL-10-deficient mice downregulated the IFN-γ secretion in CD4^+^ T cells and conferred protection against severe endotoxic shock *in vivo*. These findings demonstrate the change and notable therapeutic potential of IL-10-producing Breg cells in endotoxic shock (Tao et al., 2019). Besides, IL-35-expressing B cells have been shown to suppress inflammation by regulating Th17 cell function in both humans and mice, and injection of recombinant IL-35 or IL-35þ Breg cells into mice could resolve inflammation by suppressing effector Th1 and Th17 cell responses and regulating Treg cell responses (Shen et al., 2014; Wang et al., 2014).

## 5 Clinical applications and therapeutic potential of B cells in sepsis

### 5.1 Use of B cells as biomarkers in sepsis

Clinical studies suggest that quantitative and qualitative assessment of B cells or B-cell subsets may lead to the identification of biomarkers that can predict the diagnosis or outcome in patients with sepsis and thereby guide treatment strategies (Monserrat et al., 2013; Krautz et al., 2018). Previous clinical cohorts have shown that a reduction in circulating B cells at sepsis onset or later is associated with decreased sepsis survival, and several meta-analyses have also reached consistent conclusions (Krautz et al., 2018; Tian et al., 2019). A constructed immune index upon admission, the absolute ratio between T- and B-lymphocyte proportions (CD8/CD19 ratio), has been reported to help identify a subset of patients with a very skewed immune protection for subsequent diagnosis of sepsis during intensive care unit (ICU) stay (Frattari et al., 2018). Also, in patients with septic shock, a higher percentage (64%) of CD19^+^CD23^+^ at ICU admission appears to be a reliable biomarker of good prognosis, while within 24 h of diagnosis, the combination of immature/transitional B cells and resting memory B cells counts is significantly more predictive of 60-day mortality in sepsis than commonly used critical illness scores (Monserrat et al., 2013; Dong et al., 2020). In addition, the low percentage of CD72+/CD19+ B cells after 3–4 days after sepsis correlates with the reduction of plasma immunoglobulin M levels and is an independent predictor of 28-day mortality in patients with sepsis (Yang et al., 2020).

### 5.2 Immunoglobulin therapy in sepsis

Generally, low immunoglobulin concentrations and abnormally high free light chains levels are seen in most adult patients with sepsis; however, the association of endogenous immunoglobulin levels with the outcome of sepsis is still controversial (Shankar-Hari et al., 2017b; Akatsuka et al., 2021; Alagna et al., 2021). In addition, a single immunoglobulin component may not be as valuable as the combination of multiple immunoglobulin levels in predicting the prognosis of patients with sepsis (Bermejo-Martín et al., 2014). Nonetheless, the use of intravenous immunoglobulin (IVIg) in the clinical treatment of patients with sepsis has attracted considerable attention as IVIg represents a promising approach to modulate pro- and anti-inflammatory processes during sepsis (Perez et al., 2017). Unfortunately, although some clinical studies and animal experiments have shown that IVIg can improve the prognosis and reduce the inflammatory response in sepsis, several large randomized controlled trials (RCTs) have demonstrated that IVIg does not reduce the mortality of patients with sepsis (Werdan et al., 2007; Hagiwara et al., 2008; Werdan et al., 2008; Makjaroen et al., 2021).

Systematic reviews of RCTs suggested that IVIg may have some key limitations as a standard of therapy for patients with sepsis, including variable trial quality, uncertainty about characteristics of optimal subjects, the timing of therapy initiation, and details of drug use (Shankar-Hari et al., 2018). These limitations highlight the need for better-designed trials. Therefore, despite biological plausibility, the available evidence is not sufficient to support the widespread use of IVIg in the treatment of sepsis (Shankar-Hari et al., 2018; Cui et al., 2019). Indeed, sepsis is a heterogeneous disease, and stratifying treatment according to the sepsis subtype may better identify the beneficiary population. For example, recent studies have suggested that the number of circulating Tfh cells may be a stratification tool for IVIg therapy, as Tfh is a good indicator of not only B cell maturation but also of sepsis prognosis (Duan et al., 2020). Another study in patients with sepsis showed that simultaneous lowering of Ig and increased blood levels of light chains could serve as markers of increased risk of death and could also identify a subgroup of patients eligible for IVIg therapy (Shankar-Hari et al., 2017b).

### 5.3 Therapeutic strategies targeting B cells

**FIGURE 4:**
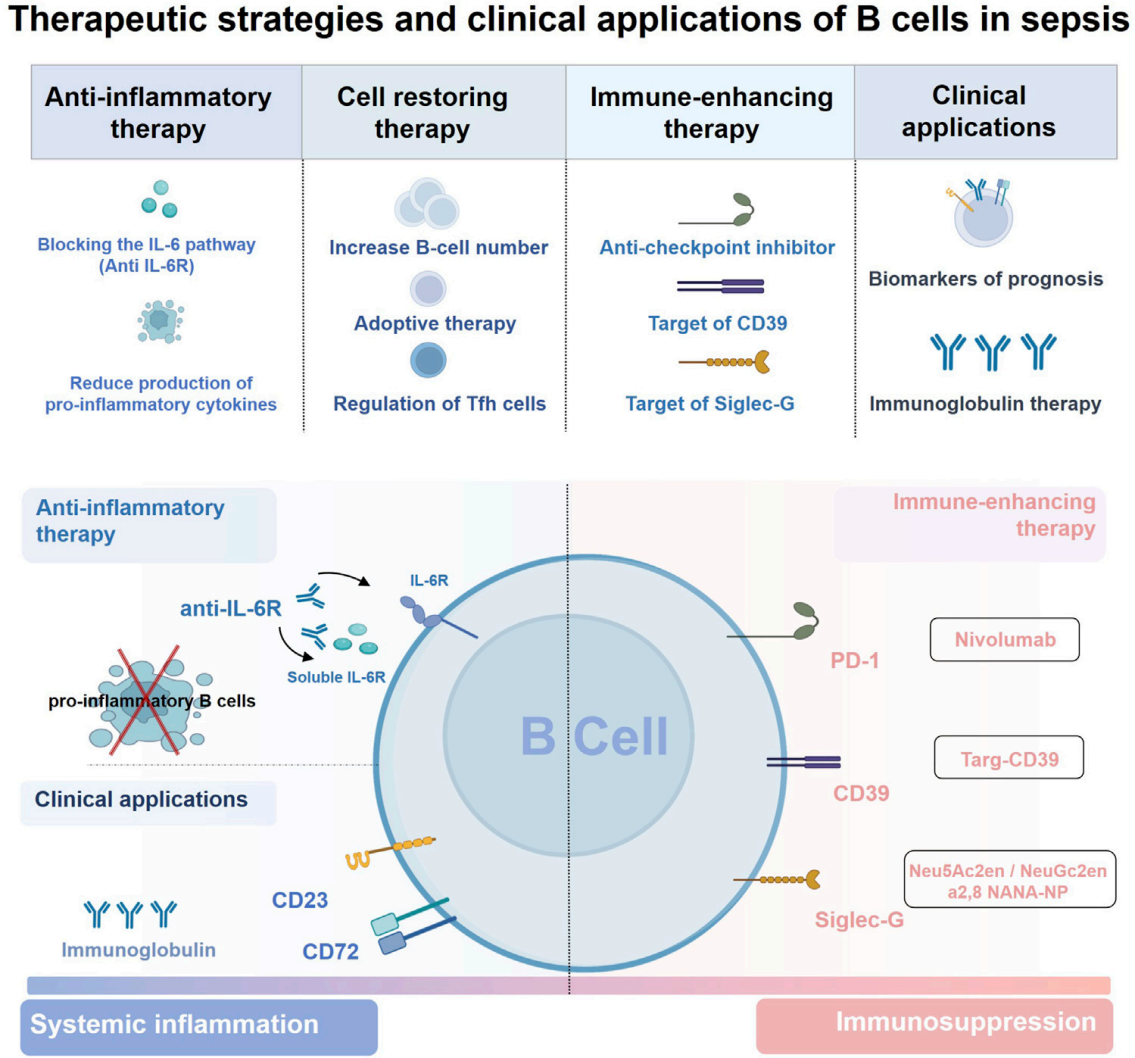
Therapeutic strategies and clinical applications of B cells in sepsis. Sepsis is characterized by concurrent excessive inflammation and immune suppression, and B cells can act as immunomodulators in the host’s immune response to sepsis. Therefore, strategies targeting B cells are considered promising for the treatment of sepsis including 1) restoration of B cell numbers and function, 2) blocking the effects of secreted cytokines or the selective depletion of pro-inflammatory B cells during systemic inflammation, 3) immune-enhancing therapy targeting B cells during sepsis immunosuppression. Of note, maintaining the balance between the number or function of proinflammatory B cells and Breg cells contributes to the regulatory role of B cells in the sepsis immune response. In addition, B cells can also be used as potential biomarkers for the diagnosis and prognosis in patients with sepsis, but the immunoglobulin therapy based on the antibody production of B cells still needs to be further optimized in clinical practice. Abbreviations: CD, cluster of differentiation; IL-6R, interleukin-6 receptor; PD1, programmed cell death 1; Siglec-G, sialic acid–binding immunoglobulin-type lectin-G; Tfh cell, T follicular helper cell.

Given that decreased B cell numbers and dysfunction are common in sepsis and are associated with poor outcomes, restoring B cell numbers and/or function and maintaining a normal peripheral B-lymphocyte pool in sepsis may help improve the outcomes (Figure 4). HSCs rejuvenation therapies, such as FOXOs and CASIN, can modulate cell survival and growth, increase the common lymphoid progenitor pool, and restore B-cell numbers (Akunuru and Geiger, 2016; de Mol et al., 2021). It could be inferred that similar HSC rejuvenation therapies and drugs may be an interesting therapeutic strategy to restore the reduced B-cell numbers in sepsis. Animal experiments have shown that the adoptive transfer of B1a cells or CD5^+^CD1dhi Breg cells can effectively reduce the inflammatory response, and prevent organ damage and severe endotoxic shock in mice with sepsis, suggesting that supplementation of specific B-cell subsets by adoptive immunotherapy could reverse the damage of B-cell reduction (Aziz et al., 2017; Aziz et al., 2018). In addition, Tfh cells are critical for GC formation and B cell maturation, and different cytokine-skewed Tfh cells differ in their ability to help B cells (Taylor et al., 2020a; Olatunde et al., 2021). Thus, regulation of Tfh cell responses may be another important target for maintaining efficient B-cell immune responses in future sepsis treatments, which deserves more attention (Olatunde et al., 2021; Ritzau-Jost and Hutloff T, 2021).

Modulating the function of pro-inflammatory B cells involved in immune response may also be a potential therapeutic strategy for sepsis. Although many anti-inflammatory strategies have failed, there may still be some hope for the use of anti-inflammatory strategies in the early stages of sepsis (Venet and Monneret, 2018). Animal models of sepsis have shown that MZ B cells contribute more to the increase in serum IL-6 levels than macrophages and exacerbate systemic inflammatory responses 4–8 h after injection of LPS or *E. coli* (Honda et al., 2016; Shibuya et al., 2017). More importantly, treatment of mice with anti-IL-6R antibody 4 h after LPS or *E. coli* injection, at which time MZ B cells start IL-6 production, significantly prolongs the survival (Shibuya et al., 2017). Therefore, blocking the IL-6 signaling pathway in MZ B cells at appropriate time points after the onset of sepsis through anti-inflammatory therapy remains a promising treatment for sepsis. B-cell depletion therapy has been shown to suppress excessive inflammation in inflammatory diseases, but this approach eliminates nearly all B cells in the body and is not suitable for sepsis (Lee et al., 2021). However, recent studies have shown that treatment of mice with plasma cell-depleted bortezomib, but not with anti-CD20-B cells ablation, prevents bleomycin-induced pulmonary fibrosis (Prêle et al., 2022). Similarly, lethally irradiated mice lacking MZ B cells were resistant to LPS-induced endotoxic shock (Honda et al., 2016). Thus, it could be concluded that selective depletion of pro-inflammatory B-cell subsets may be an optimistic therapeutic strategy in sepsis. Notably, due to the complex pathogenesis of sepsis, it is unknown whether depletion of B-cell subsets will lead to redistribution of B cells and/or compensatory effects of other non-B cells. Nevertheless, selective depletion of specific pro-inflammatory B-cell subsets during an excessive inflammatory response in sepsis remains a promising therapeutic option that warrants further exploration.

Therapeutic strategies aimed at enhancing immune function during the immunosuppressive phase of sepsis are being widely explored, but few studies have focused on B cells (Torres et al., 2022). Similar to studies on T cells, blocking immune checkpoint inhibitors on B cells during immunosuppression may be a potential therapeutic strategy for sepsis. The aberrant activation of the PD-1/PD-L1 immune checkpoint pathway is a major cause of immune paralysis in sepsis (Nakamori et al., 2020). It has been highlighted that the expression of these checkpoint regulators is increased in B cells in patients with sepsis, particularly in the memory B subset, which is related to poor patient outcomes (Wilson et al., 2018). Importantly, animal models have demonstrated enhanced antibody production and improved survival in PD-1 knockout mice in CLP mice, and several phase 1B/2 clinical trial studies have confirmed that nivolumab, a PD-1 monoclonal antibody, has good safety and tolerability in sepsis patients (Huang et al., 2009; Hotchkiss et al., 2019a; Hotchkiss et al., 2019b; Watanabe et al., 2020). These data suggest that anti-checkpoint inhibitor therapy is promising for improving immunosuppression in sepsis, with some benefit from reversing B-cell dysfunction and increasing antibody production (although it is uncertain to what extent). However, further clinical trials are still needed. Reversal of exhausted-like/immunoregulatory B-cell responses is another immunostimulatory therapeutic strategy for sepsis. Both septic patients and animals exhibit enhanced accumulation of CD39^+^ plasmablasts, an important driver of sepsis-induced immunosuppression, which is associated with impaired bacterial killing and poor outcomes (Nascimento et al., 2021; Zhou et al., 2021). A preclinical study showed that the recombinant protein targ-CD39, which targets CD39 to activated platelets, can reduce systemic inflammation and improve survival in sepsis (Granja et al., 2019). Therefore, this innovative therapeutic approach holds promise for selective targeting of CD39 on B cells and for inhibiting the function of CD39^+^ plasmablasts to hinder sepsis-induced immunosuppression. Besides, sialic acid–binding immunoglobulin-type lectin-G (Siglec-G) is another target molecule that is mainly expressed in B-1 cells (Royster et al., 2021a). Downregulation of Siglec-G expression on B1a cells predisposes them to a proinflammatory phenotype and hinders its immunoregulatory function in sepsis, which ultimately promotes death in septic mice (Royster et al., 2021b). Thus, the preservation of Siglec-G on B1a cells is considered a novel therapeutic approach in sepsis. Several drugs that target Siglec-G, including sialidase inhibitors (Neu5Ac2en, NeuGc2en) and sialic acid-mimicking nanoparticles (a2,8 NANA-NP), are effective in maintaining the immunomodulatory status of B1a cells and providing protection in polymicrobial sepsis (Chen et al., 2011; Spence et al., 2015). And this provides further application prospect for B-cell based therapy of sepsis.

## 6 Conclusion and perspectives

The role of the immune system is central in the development and progression of sepsis. Within the complex network of immunity in sepsis, B cells possess powerful properties. B lymphopenia is generally present in adult sepsis, with dynamic alterations including increased dysfunction, cellular exhaustion, and redistribution of B-cell subsets, which greatly restricts the involvement of B cells in sepsis immune response. The roles of B cells in sepsis are multiple and complex, depending on how subsets and their functions are targeted, and the balance between Breg cells and pro-inflammatory B cells may act as a regulatory switch for the immune system. Indeed, since B cells play significant roles in both innate and adaptive immune responses, immunomodulatory therapy targeting B cells may represent a two-pronged approach.

The following remain to be addressed: the dynamic changes of B cells in children and elderly patients with sepsis need to be further clarified; it is not fully understood how the functions of different B-cell subsets are regulated and coordinated during sepsis; the application of B cells in the clinical treatment of sepsis, including the screening of sepsis subtypes suitable for IVIg, needs to be further explored. In addition, the role of B cells in immune responses could also be regulated by components of innate immunity, such as neutrophil extracellular traps (NETs) (Costa et al., 2019). Neutrophil extracellular traps (NETs) are released by neutrophils and exert antimicrobial functions during infections, and there is increasing evidence that they also regulate B-cell activation, differentiation, and associated immune responses (Costa et al., 2019; Karmakar,, 2021). NETs induce BAFF secretion and directly activate B cells through an antigen non-specific mechanism (Cervantes-Luevano et al., 2018; Dömer et al., 2021). Also, NETs could stimulate NET-specific self-reactive B cells through exposure to the BCR, and promote B-cell differentiation and autoantibody production (Gestermann et al., 2018). As such, the regulatory effect of NETs on B cells during sepsis is worthy of further attention. Besides, a recent study showed that B-cell metabolic adaption is characterized by an increase in glycolysis and respiration over time in sepsis, and the metabolic upregulation goes together with a shift toward antibody-producing subtypes (Schenz et al., 2020). This finding underscores the need for a personalized understanding of B-cell molecular and immunological variation, which may provide a new way to better understand the mechanisms of sepsis immune disorders and explore therapeutic targets of B cells in sepsis.

## Glossary

ADCC: antibody-dependent cellular cytotoxicity
ADCP: antibody-dependent cellular phagocytosis
APCs: antigen-presenting cells
BAFF: B-cell activating factor
BCR: B cell receptor
Breg: regulatory B cell
CD: cluster of differentiation
CDC: complement-dependent cytotoxicity
CLP: cecal ligation and puncture
DC: dendritic cell
*E*.*coli*: *Escherichia coli*
Fas: cell surface death receptor
FasL: cell surface death receptor ligand
FO: follicular
GCs: germinal centers
GM-CSF: granulocyte macrophage-colony stimulating factor
HSCs: hematopoietic stem cells
ICU: intensive care unit
IFN-γ: interferon-γ
Ig: immunoglobulin
IL: interleukin
IRA: innate response activator
IVIg: intravenous immunoglobulin
LPS: lipopolysaccharide
MHC-II: major histocompatibility complex class II
MZ: marginal zone
NETs: neutrophil extracellular traps
NK cell: natural killer cell
PD1: programmed cell death 1
PDL1: programmed cell death 1 ligand 1
*S. aureus*: *Staphylococcus aureus*
Siglec-G: sialic acid–binding immunoglobulin-type lectin-G
Tfh: T follicular helper
TGF-β: transforming growth factor-β
Th: T helper cell
TLRs: Toll-like receptors
TNF-α: tumor necrosis factor-α
Treg: regulatory T cell
